# Long‐term inflammatory rosacea management with subantibiotic dose oral doxycycline 40 mg modified‐release capsules once daily

**DOI:** 10.1111/dth.15180

**Published:** 2021-12-02

**Authors:** James Q. Del Rosso, Sam Brantman, Hilary Baldwin

**Affiliations:** ^1^ JDR Dermatology Research Thomas Dermatology Las Vegas Nevada USA; ^2^ Clinical Research and Strategic Development Advanced Dermatology and Cosmetic Surgery Maitland Florida USA; ^3^ Adjunct Clinical Professor (Dermatology) Touro University Nevada Henderson Nevada USA; ^4^ Galderma Laboratories, L.P. Fort Worth Texas USA; ^5^ The Acne Treatment and Research Center Brooklyn New York USA; ^6^ Department of Dermatology Rutgers Robert Wood Johnson Medical Center New Brunswick New Jersey USA

**Keywords:** metronidazole 1%, moderate or severe inflammatory lesions of rosacea, randomized controlled trial, remission, subantibiotic dose doxycycline

## Abstract

An important goal of effective rosacea treatment is to maximize the duration of remission. This was a two‐part study. Part 1 was a multicenter, open‐label, 12‐week study in which adults with moderate or severe inflammatory lesions (papules and pustules) of rosacea received subantibiotic dose oral doxycycline 40 mg modified release (SDD_40_) and topical metronidazole gel 1%. Part 2 was a multicenter, randomized, double‐blind, placebo‐controlled, 40‐week study in which successfully treated subjects received once‐daily SDD_40_ or placebo capsules. The primary objective was to assess relapse and efficacy during long‐term use of SDD_40_ versus placebo. Relapse was defined as a return to baseline investigator global assessment (IGA) or lesion count, or any other necessary change in treatment. Part 1 enrolled 235 subjects. Sixty‐five subjects in the SDD_40_ treatment group and 65 subjects in the placebo group met the definition of treatment success at week 12, and were included in the Part 2 analysis. At the end of Part 2, half as many subjects in the SDD_40_ group had relapsed compared to the placebo group (13.8% [*n* = 9] vs. 27.7% [*n* = 18], *p* < 0.05). Significant differences in the median change in inflammatory lesion counts were also observed (*p* < 0.05). Adverse events (AEs) were generally mild–moderate in severity, and most were not treatment‐related. Stinging/burning responded with more improvement in subjects treated with SDD_40_. After 52 weeks of once‐daily treatment, subantibiotic dose doxycycline significantly reduced the relapse rate and inflammatory lesion counts in subjects with moderate‐to‐severe inflammatory rosacea.

## 1 INTRODUCTION

Rosacea is a dynamic disease, with affected individuals experiencing recurrent cycles of exacerbation and remission over their lifetime.^1^ While the underlying disease is not curable, rosacea flares are managed in clinical practice using a combination of patient education, trigger avoidance, skincare, and rational medication selection.^2^ Importantly, the fundamental goals of therapy are to achieve clearance of the current rosacea flare to maximize the duration of remission after successful control with initial therapy.

Topical metronidazole and subantibiotic dose doxycycline, administered as a 40 mg modified‐release (MR) capsule once daily, have been reported to be an effective combination therapy for a flare of papulopustular rosacea.^3^ Initially referred to in the literature as subantimicrobial dose doxycycline, the designated doxycycline 40 mg MR capsule delivers 30 mg of immediate‐release and 10 mg delayed‐release (via beads) doxycycline in the gastrointestinal tract; this is more accurately referred to as *subantibiotic dose doxycycline* (hereafter called SDD_40_), as pharmacokinetic and microbiologic data strongly support negligible selection pressure on bacteria with avoidance of antibiotic resistance.^3^, ^4^ Topical metronidazole is a highly tolerable, nitroimidazole derived, antiprotozoal, antibacterial, antioxidative, and anti‐inflammatory agent, and it has been proposed that metronidazole reduces the signs and symptoms of rosacea primarily through its anti‐inflammatory and antioxidative activity.^5^, ^6^ SDD_40_ is the only oral therapy approved by the United States Food and Drug Administration (FDA) for rosacea, specifically for the treatment of the inflammatory lesions (papules and pustules). Plasma concentrations of SDD_40_ remain below the threshold necessary for antibiotic activity; instead, data support that doxycycline primarily targets inflammatory lesions (papules and pustules) of rosacea through anti‐inflammatory activity.^4^, ^7^, ^8^, ^9^ Doxycycline may influence inflammation in rosacea through a variety of mechanisms, including modulation of the cathelicidin pathway, matrix metalloproteinases, serine protease activity, neutrophil chemotaxis, phospholipase A2, nitric oxide, interleukin (IL)‐6, and reactive oxygen species activity.^9^ The anti‐inflammatory activity of SDD_40_ has been demonstrated to effectively and safely reduce the inflammatory lesions of rosacea in large, randomized, and community‐based clinical trials.^7^, ^8^, ^9^, ^10^ In the pivotal studies, SDD_40_ was used as monotherapy compared with placebo.^9^ Treatment with a combination of topical metronidazole gel 1% and SDD_40_ has been shown to produce a more rapid and greater reduction of inflammatory lesions compared with topical metronidazole monotherapy; however, previous investigations did not examine the long‐term efficacy of SDD_40_ in sustaining remission of rosacea presenting with inflammatory lesions.^11^

Extending the duration of remission after successful therapy to control a flare is a fundamental goal of rosacea management. This study evaluated the efficacy of SDD_40_ monotherapy in extending the duration of rosacea remission.

## 2 METHODS

### 2.1 Study design

The primary objective of this two‐part study was to evaluate the rate of relapse and efficacy of long‐term treatment with SDD_40_ or placebo after an initial successful 12‐week regimen of SDD_40_ and topical metronidazole gel 1% in patients with inflammatory lesions of rosacea. In both parts, subjects were provided with a gentle skin cleanser and sun‐protective moisturizer with SPF 15 (Cetaphil® Gentle Skin Cleanser and Cetaphil® Daily Facial Moisturizer SPF 15; Galderma Laboratories L.P., Fort Worth, TX).

Part 1 was a multicenter, open‐label study. Eligible subjects were treated once daily in the morning for 12 weeks with SDD_40_ (Oracea® Capsules; Galderma Laboratories L.P., Fort Worth, TX) given orally and metronidazole 1% (MetroGel®, 1%; Galderma Laboratories L.P., Fort Worth, TX) applied topically. Four evaluations were conducted at baseline and at 4, 8, and 12 weeks. At each visit, inflammatory lesion counts, safety and tolerability assessments, and investigator global assessment (IGA) were recorded along with any changes in concomitant medication use, adverse events (AEs), and subject diary review. At week 12, subjects were evaluated for Part 2 eligibility.

**FIGURE 1:**
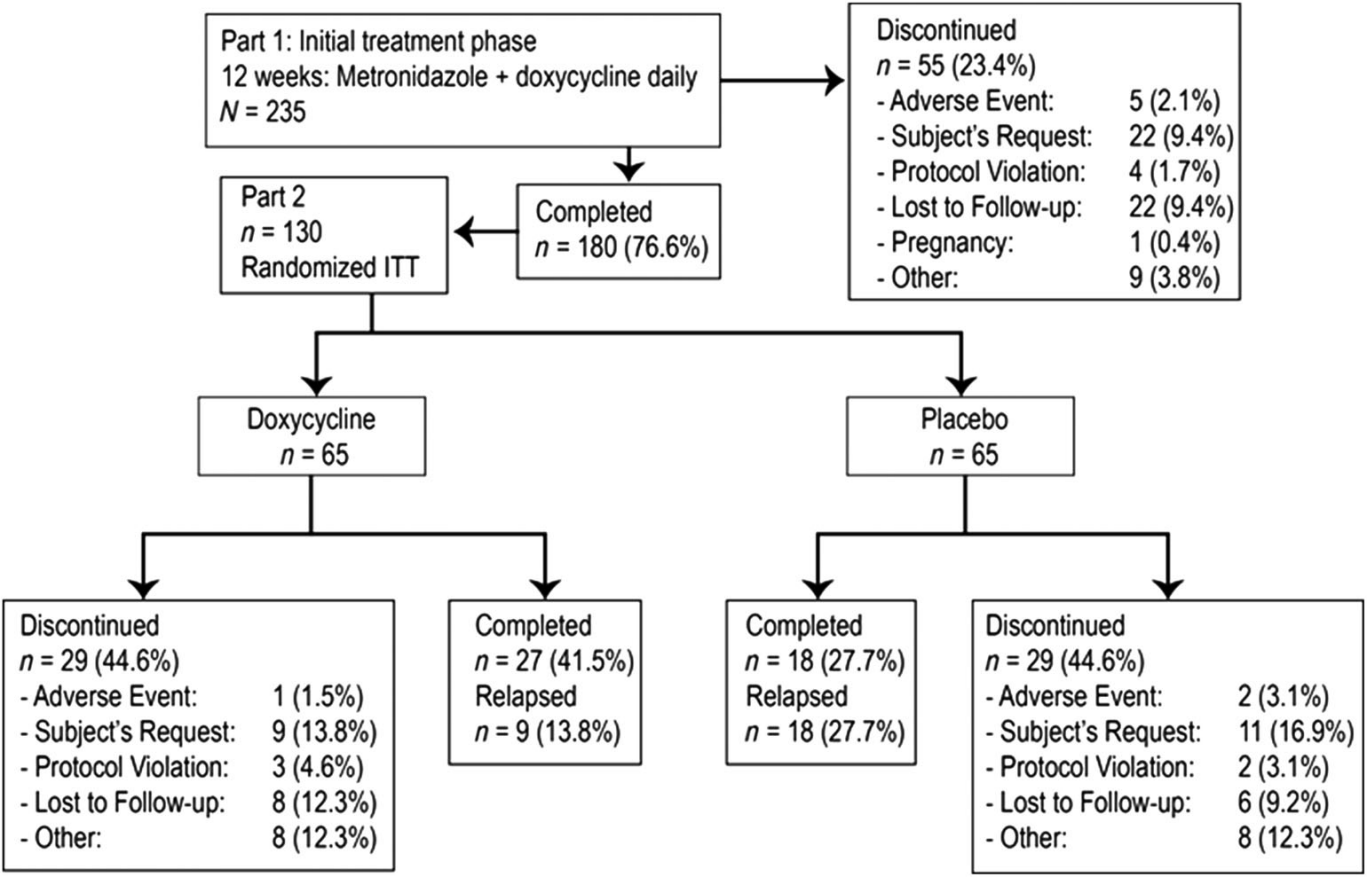
Study design and subject disposition

Part 2 was a multicenter, randomized, double‐blind, placebo‐controlled study in which eligible subjects were treated with SDD_40_ or placebo capsules once daily for up to an additional 40 weeks of treatment. Subjects with IGA scores of 0 or 1 (clear or near clear), or at least a 2‐grade IGA improvement (from severe [IGA 4] to at least mild [IGA 2]) were equally randomized (1:1) into each treatment arm. Subjects who relapsed during Part 2 were discontinued from the study. Relapse was defined as a return to the baseline lesion count, return to the baseline IGA, or any clinical condition that in the judgment of the investigator warranted a change in rosacea treatment. Subjects were evaluated at weeks 4, 8, 12, 16, 20, 24, 28, 32, 36, and 40. The study design is illustrated in Figure 1. The total study duration of Part 1 (12 weeks) and Part 2 (40 weeks) was 52 weeks.

### 2.2 Subjects

Men and women aged 18 to 80 years (inclusive) were included in the study if they had a diagnosis of inflammatory lesions of rosacea (with 11 to 40 papules or pustules) and an IGA of moderate or severe (IGA 3 or IGA 4, respectively, using a 5‐point scale, 0–4; Table 1). Women with childbearing capability were required to have a negative urine pregnancy test at baseline and agreed to practice an effective method of contraception over the study duration. If a hormone‐based contraceptive was used, the subject had to be on a stable dose for 90 days before baseline and was required to maintain the same dose throughout the study. Concurrent medications were allowed if the investigator did not believe they would interfere with study results. Medications and doses were required to remain stable for 90 days prior to baseline, and not expected to change during the study. The safety population included all subjects enrolled in Part 1 of the study, and the intent‐to‐treat (ITT) population was defined as all subjects who were randomized with study drug dispensed. The ITT population was used for efficacy analysis.

**TABLE 1:** Investigator global assessment (IGA)

IGA		Definition	Guideline
0	Clear	No signs or symptoms present	Skin is completely clear of inflammatory lesions
1	Near clear	One or two papules	1 or 2 small, non‐inflammatory papules
2	Mild	Some papules/pustules	3–10 papules/pustules
3	Moderate	Moderate number of papules/pustules	11–19 papules/pustules
4	Severe	Numerous papules/pustules; nodules	≥20 papules/pustules and nodules

Subjects were excluded if they met any of the following criteria: pregnant or planning a pregnancy; allergy or hypersensitivity to any study drug component; other active facial dermatoses that could interfere with the study assessments; use of medications/therapies that might interfere with study results; phymatous rosacea; the presence of any risks described in the precautions, warnings, and contraindications of the study drugs; or high likelihood of extensive ultraviolet (UV) exposure.

At the end of Part 1, subjects who met eligibility requirements were randomized into Part 2. Subjects eligible for Part 2 were those who met one of the following criteria: achieved an IGA score of clear (0) or near clear (1); achieved at least a 2‐grade improvement from their baseline IGA score. Subjects were removed from the study if they experienced a relapse.

### 2.3 Subject evaluation

Evaluations performed at baseline and at each study visit included inflammatory lesion counts (papules and pustules) and assessment of rosacea severity by IGA (Table 1). Treatment success was defined as a two‐grade improvement in IGA, or an IGA score of clear or near clear.

Skin tolerability was assessed using subject satisfaction questionnaires at baseline and the end of parts 1 and 2; AE monitoring and reporting was conducted throughout the study via questioning of the subject.

### 2.4 Statistical methods

The sample size required for this investigation was estimated using the relapse rate and percentage of subjects who experienced a two‐grade improvement in IGA after using SDD_40_ and topical metronidazole gel 1% for 12 weeks, based on previous studies.^3^, ^10^ For Part 2, it was estimated that a minimum of 80 subjects (40 subjects per treatment arm) would be required to show 5% significance with at least 80% power.

Missing data were imputed using mixed models. Subject disposition, demographics, and baseline characteristics were summarized by descriptive statistics. Categorical variables were summarized by frequency and percentage for each response category. Continuous variables were summarized using descriptive statistics for the data collected at each visit.

The study hypothesis was that subjects treated with SDD_40_ would experience a longer time to relapse when compared to the placebo group. Therefore, subjects were removed from the study once they experienced a relapse. Change from baseline in mean inflammatory lesions was analyzed by repeated‐measures analysis of variance. Last observation carried forward (LOCF) was used to impute missing efficacy data. Significance was calculated using two‐sample Wilcoxon and Cochran–Mantel Haenszel chi‐square tests stratified by investigational site, with a *p*‐value ≤0.05 considered to be significant.

The relationship between the IGA score and inflammatory lesion counts at week 12 in both the SDD_40_ and placebo groups was analyzed.

**FIGURE 2:**
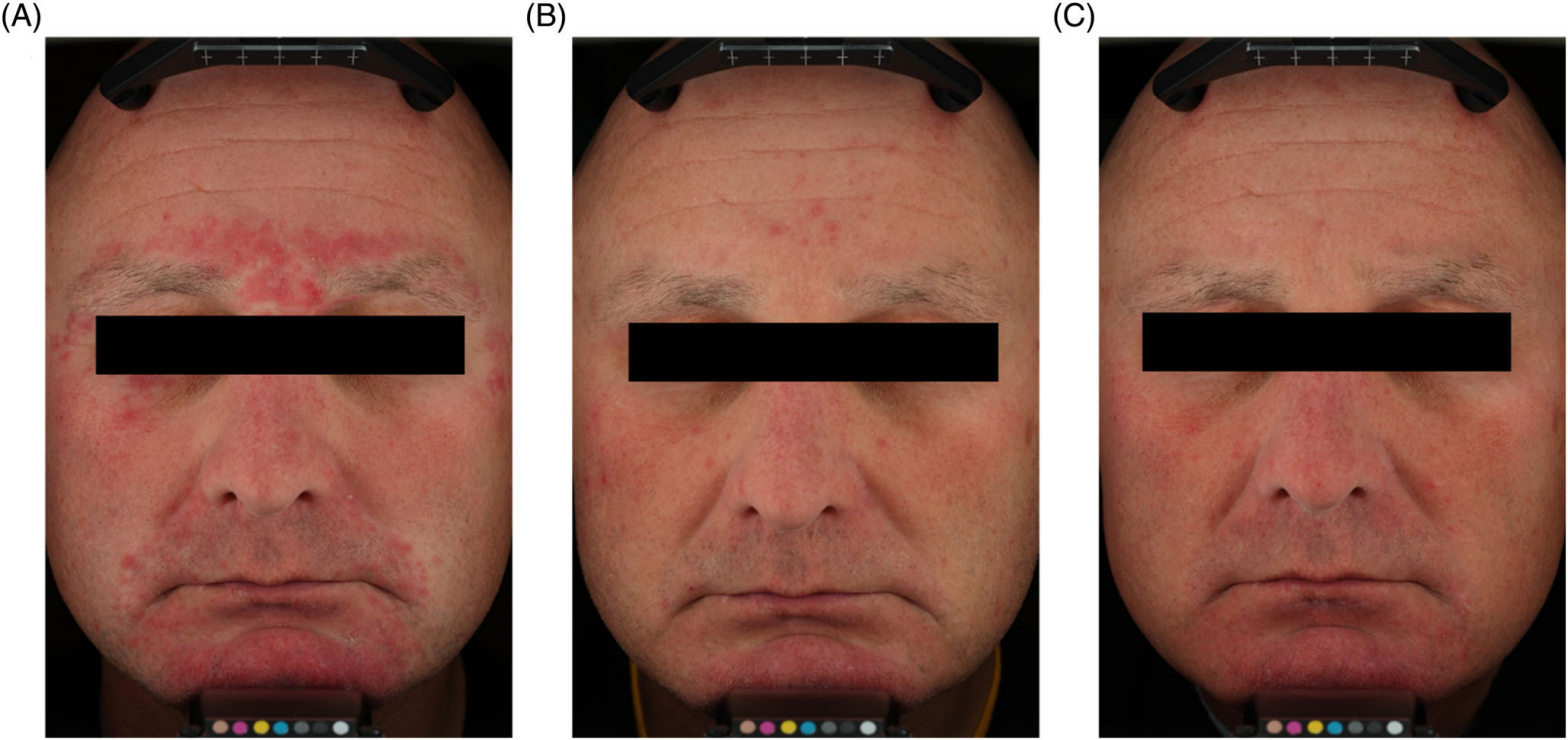
Subject photographs of treatment success and long‐term management of inflammatory lesions of rosacea with subantibiotic dose oral doxycycline 40 mg. Subject pictured is a 43 year‐old male with severe inflammatory rosacea at baseline (A), successfully treated with topical metronidazole 1% and subantibiotic dose oral doxycycline, and assessed to have an IGA score of 0 (clear) at the week 12 visit (B). The subject was then randomized to receive subantibiotic dose oral doxycycline for an additional 40 weeks of treatment. The subject completed the 40‐week long‐term management part without relapse and was assessed to have an IGA score of 1 (near clear) at the final study visit (C)

Study protocols were approved by independent ethics committees and conformed to the Declaration of Helsinki. All subjects who participated in this trial were fully informed about the study in accordance with applicable regulations. Written informed consent was received from the patient for the use of images and publication of his case details (Figure 2).

## 3 RESULTS

### 3.1 Enrollment and demographics

Most enrolled subjects were white women of non‐Hispanic or Latino ethnicity. The mean age of enrolled subjects was 47.4 years, and subjects had a 5‐year history of rosacea on average. Subject demographics were similar between groups (Table 2).

**TABLE 2:** Baseline demographics and clinical characteristics

		Part 2	
	Part 1	Subantibiotic dose oral doxycycline (SDD_40_)	Placebo
Enrolled	235	65	65
Gender, *n* (%)			
Male	66 (28.7%)	20 (30.8%)	24 (36.9%)
Female	164 (71.3%)	45 (69.2%)	41 (63.1%)
Ethnicity, *n* (%)			
Hispanic or Latino	44 (19.1%)	12 (18.5%)	14 (21.5%)
Not Hispanic or Latino	186 (80.9%)	53 (81.5%)	51 (78.5%)
Race, *n* (%)			
White	217 (94.3%)	63 (96.9%)	63 (96.9%)
Black/African American	7 (3.0%)	0 (0.0%)	1 (1.5%)
Asian	1 (0.4%)	1 (1.5%)	0 (0.0%)
Other	5 (2.2%)	1 (1.5%)	1 (1.5%)
Age, mean years (*SD*)	47.4 (12.9)	47.2 (13.2)	51.7 (12.8)
Skin type (*n*, %)			
Dry	40 (17.4%)	12 (18.5%)	15 (26.8%)
Normal	50 (21.7%)	9 (13.8%)	8 (14.3%)
Oily	38 (16.5%)	9 (13.8%)	12 (21.4%)
Combination	102 (44.3%)	35 (53.8%)	21 (37.5%)
Fitzpatrick skin type (*n*,%)			
I	16 (7.0%)	2 (3.1%)	3 (5.4%)
II	93 (40.4%)	26 (40.0%)	25 (44.6%)
III	64 (27.8%)	22 (33.8%)	14 (25.0%)
IV	42 (18.3%)	11 (16.9%)	11 (19.6%)
V	15 (6.5%)	4 (6.2%)	3 (5.4%)
VI	0 (0.0%)	0 (0.0%)	0 (0.0%)
Baseline IGA, *n* (%)			
Clear	0	34 (52.3%)	32 (49.2%)
Near clear	0	22 (33.8%)	24 (36.9%)
Mild	0	9 (13.8%)	9 (13.8%)
Moderate	149 (64.8%)	0	0
Severe	81 (35.2%)	0	0
Missing	0	0	0
Baseline inflammatory lesions			
Mean	19.8	1.2	1.3
*SD*	7.4	1.7	1.9
Median	18.0	0.0	1.0
Range (min, max)	(11, 40)	(0, 8)	(0, 10)

At Part 1 baseline, 235 subjects were enrolled with IGA scores of moderate (*n* = 149, 64.8%) or severe (*n* = 81, 35.2%), and a mean of ~20 inflammatory lesions (Table 2). Two‐hundred and thirty subjects received treatment and were included in the ITT population. Subjects who met the definition of treatment success (clear, near clear, or 2‐grade improvement) at week 12 were included in Part 2. One‐hundred and eighty subjects completed Part 1, and 130 of these subjects were randomized into Part 2, receiving monotherapy with either SDD_40_ once daily (*n* = 65) or placebo capsule once daily (*n* = 65) after first achieving treatment success on or before week 12 in Part 1 (Figure 1). No topical therapy was utilized during Part 2 of the study.

### 3.2 Part 1 efficacy

At baseline, all subjects had IGA scores of moderate (64.8%) or severe (35.2%). At the end of Part 1, 50.9% of all subjects enrolled in the study met the definition of treatment success. The mean number of inflammatory lesions at baseline in Part 1 was ~20. At the end of Part 1, after 12 weeks of treatment with SDD_40_ and topical metronidazole gel 1%, subjects had a mean of 5 inflammatory lesions, with a median change from baseline of −14.0 inflammatory lesions. Of the 180 subjects who completed Part 1, after 12 weeks of treatment, 130 met the definition of treatment success (72.2%).

### 3.3 Part 2 efficacy

**FIGURE 3:**
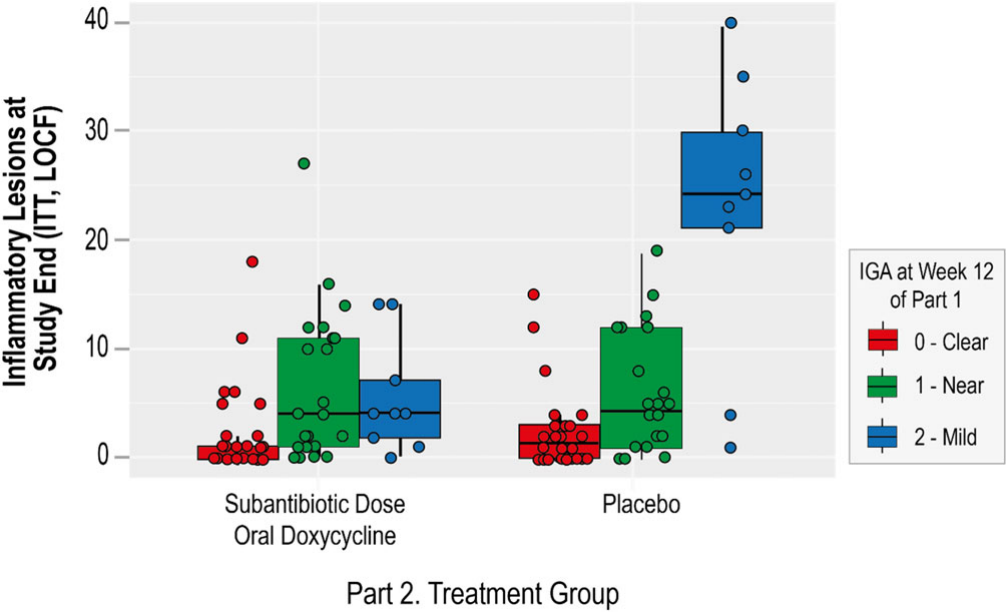
End of study lesion counts in treatment and placebo groups correlate with week 12 IGA. The end of study inflammatory lesion counts of subjects in the subantibiotic dose doxycycline and placebo treatment groups were analyzed according to their IGA scores at the part 1 week 12 assessment (0 [clear], 1 [near clear], and 2 [mild]). After 40 weeks of treatment, subjects who entered part 2 with an IGA score of 2 (mild) maintained a significantly lower number of inflammatory lesions in the subantibiotic dose doxycycline group, when compared with the placebo treatment group (*p* = # [*r* = 0.3])

During all Part 2 assessments, the mean change in inflammatory lesion counts remained superior in the SDD_40_ group as compared with the placebo group (*p* < 0.05). Subjects who entered Part 2 with higher IGA scores displayed significantly higher lesion counts at the end of the study in the placebo group (Figure 3).

### 3.4 The rate of relapse in Part 2

**FIGURE 4:**
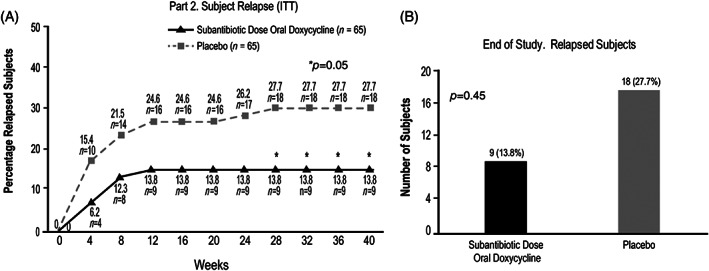
Rate of relapse during part 2: (A) Relapsed subjects over time (ITT) and (B) relapsed subjects at study end

The primary objective of Part 2 was the evaluation of relapse. After 40 weeks of treatment (week 52 of the study, Part 1 inclusive), half as many subjects in the SDD_40_ group had relapsed when compared with subjects in the placebo group (13.8% [*n* = 9] vs. 27.7% [*n* = 18], respectively; *p* < 0.05; Figure 4B). Statistically significant differences were seen in favor of the SDD_40_ group from week 28 of Part 2 until the study end (*p* < 0.05; Figure 4A,B).

Relapse occurred more quickly, with greater frequency, and at a larger number of study visits in the placebo group, with 10 subjects experiencing relapse by week 4 of Part 2 in the placebo group, compared with 4 in the SDD_40_ group. In the SDD_40_ group, no additional relapse events occurred after the first 12 weeks of Part 2 treatment. In contrast, individuals were discontinued from the placebo group due to relapse at both the week 24 and week 28 visits (Figure 4A). In summary, relapse occurred in twice as many subjects in the placebo group compared with the SDD_40_ group (18 vs. 9, respectively; Figure 4B).

### 3.5 Safety

Both treatments were safe and well‐tolerated in this study. Adverse events were generally mild or moderate in severity during both parts of the study. A similar number of AEs were reported in both the SDD_40_ and placebo groups (12 subjects in each group), and no serious or gastrointestinal AEs were considered to be treatment‐related.

For respondents to satisfaction questionnaires, an improvement in stinging/burning was seen at week 12 (end part 1 [ITT]): 81.8% of subjects had no stinging/burning or were not bothered by it (trending up from 49.6% at baseline). Furthermore, a large proportion of the improvement in stinging/burning seen after successful treatment with combination oral and topical therapy (end of Part 1) was maintained with monotherapy alone (through the end of Part 2). This improvement was greater in subjects treated with SDD_40_: 88.1% for SDD_40_ and 57.4% for placebo had no stinging/burning or were not bothered by it.

## 4 DISCUSSION

The current study demonstrated that, among subjects given successful combination therapy to treat a flare of inflammatory lesions of rosacea, long‐term treatment with SDD_40_ used as monotherapy extended the duration of remission, providing continued relief from rosacea signs and symptoms.

Due to the chronic nature of rosacea, effective treatment is important to avoid the long‐term consequences of flares and the potential of worsening rosacea severity.^2^ Rosacea may be incurable, but it is possible to consistently improve the physical appearance and quality of life of many affected individuals, and to forestall potential progression of fixed manifestations of rosacea by prolonging the time to relapse.^7^, ^12^, ^13^, ^14^ Rosacea therapy may be optimized by using combination regimens that incorporate different and complementary modes of action that target multiple pathophysiologic pathways of rosacea. Because rosacea is characterized by episodic and unpredictable flares, an effective and safe therapy that extends the duration of remission is highly valuable.

SDD_40_ is the only FDA‐approved oral therapy for the treatment of the inflammatory lesions of rosacea, with an extensive body of data supporting its safety and efficacy; a comprehensive Cochrane review of rosacea treatments rated the published evidence supporting the use of SDD_40_ for the inflammatory lesions of rosacea as “high.”^4^, ^7^, ^8^, ^9^, ^10^, ^15^, ^16^, ^17^, ^18^ It is thought that the primary mode of action of doxycycline in rosacea is via the inhibition of multiple inflammatory processes, however, use of both short‐term and/or long‐term administration of antibiotic dose doxycycline or other antibiotics increases the risk of antibiotic resistance.^4^, ^18^, ^19^ SDD_40_ has demonstrated anti‐inflammatory activity in rosacea, and avoids antibiotic selection pressure and bacterial resistance.^4^, ^7^, ^9^, ^10^ In the current two‐part study, SDD_40_ was safe and tolerable, and after prior control of a rosacea flare with initial combination therapy, this treatment as monotherapy reduced the rate of relapse and papulopustular lesion counts when compared with placebo after 40 weeks of treatment.

Results of this study emphasize the importance of comprehensive rosacea management inclusive of controlling flares and sustaining remission. Twice as many subjects treated with placebo relapsed as compared with SDD_40_, suggesting that use of SDD_40_ effectively sustains the control of rosacea. Furthermore, even among subjects who did not relapse, those who were not completely clear at the beginning of Part 2 exhibited a notably significant increase in lesion number at end of study in the placebo group. Likewise, even among subjects who did not relapse and were completely clear at the beginning of Part 2, there was a trend towards increased inflammatory lesion numbers in the placebo group. Previous studies have established that SDD_40_ significantly improves efficacy compared to topical therapy alone, supporting the use of multiple strategies to simultaneously target the signs and symptoms of rosacea.^3^, ^4^, ^7^, ^8^ Indeed, rosacea not only carries a high burden for visible manifestations, but also invisible symptoms such as stinging and burning that in our study were decreased with SDD_40_ monotherapy after successful treatment with combination therapy.

In this study, we chose a combination of SDD_40_ and topical metronidazole as the Part 1 combination therapy. Topical metronidazole has a long and established history as a rosacea therapy, and is still considered a safe and effective treatment for rosacea presenting with inflammatory lesions.^6^ Other topical therapies for rosacea presenting with inflammatory lesions are available and FDA‐approved, including azelaic acid and ivermectin.^7^ A clinical study as well as a meta‐analysis indicate that ivermectin may be a superior topical agent for the treatment of inflammatory lesions of rosacea.^20^, ^21^ Ivermectin is a newer topical agent that exhibits both anti‐inflammatory and anti‐parasitic mechanisms, demonstrates favorable efficacy, skin tolerability and safety, and maintains more prolonged remission when compared with topical metronidazole.^2^, ^6^, ^20^, ^21^, ^22^, ^23^, ^24^, ^25^ Thus, ivermectin may be considered as a preferred topical of choice to pair with SDD_40_ in future studies of long‐term combination therapy for rosacea presenting with inflammatory lesions.

In conclusion, after successful once‐daily treatment with topical metronidazole and subantibiotic dose doxycycline to control a rosacea flare, 40 weeks of subantibiotic dose doxycycline monotherapy reduced both the relapse rate and inflammatory lesions versus placebo. These data support that subantibiotic dose doxycycline is a rational, safe, and effective long‐term therapy for inflammatory lesions of rosacea.

## CONFLICT OF INTEREST

Sam Brantman, PharmD is an employee of Galderma Laboratories.

## Data Availability

The data that support the findings of this study are available on request from the corresponding author. The data are not publicly available due to privacy or ethical restrictions.
